# Provision of chlamydia testing, and training of primary health care staff about chlamydia testing, across four European countries

**DOI:** 10.1186/1471-2458-14-1147

**Published:** 2014-11-05

**Authors:** Anneli Uusküla, Ellie J Ricketts, Claire Rugman, Ruth R Kalda, Hans Fredlund, Johan Hedlund, Brigitte Dunais, Pia P Touboul, Cliodna McNulty

**Affiliations:** Department of Public Health, University of Tartu, Tartu, Estonia; Public Health England, Primary Care Unit, Microbiology, Gloucestershire Royal Hospital, Gloucester, UK; Polyclinic, University of Tartu, Tartu, Estonia; Clinical Microbiology, Örebro University Hospital, Örebro, Sweden; Office for Communicable Disease Control, Uppsala County Council, Uppsala, Sweden; Nice University Hospital, Nice, France

**Keywords:** Chlamydia, Testing, Training, General practice, Europe

## Abstract

**Background:**

The objectives of this study were to describe and compare chlamydia testing provided by general practitioners (GPs) in four selected European countries with well-developed primary health care systems and high reported chlamydia rates; we aimed to compare contrasting countries where chlamydia testing is provided by GPs (England, Sweden) with countries where primary care chlamydia testing is absent or very limited (France, Estonia).

**Methods:**

For data generation a structured questionnaire was developed and secondary data sources were searched. The questionnaire developed by the research team allowed a systematic approach to analysing chlamydia care (including testing in general practice) and the gathering of relevant data.

**Results:**

There were no significant differences in the burden of the disease or the type of general practice care provision in the study countries. In all four countries, testing for chlamydia (with nucleic acid amplification test, NAAT) is available in the public sector, a substantial proportion (>60%) of young people aged 16–25 years visit their general practitioner (GP) annually, and reimbursement for chlamydia testing costs to the relevant parties (GPs in England, Sweden and Estonia; and patients in France) by the national health insurance system or its equivalent.

In countries where chlamydia testing is provided by GPs (England, Sweden) a national strategy or plan on STI control that specifically mentions chlamydia was in force, chlamydia care guidelines for GPs were in place and STI management was more firmly established in the GP residency training curriculum, either formally (England) or informally (Sweden), than in the other countries.

**Conclusion:**

Future research on the effectiveness of chlamydia screening (also in the context of general practice care) and program provision should reflect national needs and the prevention of complications.

**Electronic supplementary material:**

The online version of this article (doi:10.1186/1471-2458-14-1147) contains supplementary material, which is available to authorized users.

## Background

As the most frequently reported notifiable communicable disease in Europe and North America, chlamydia is the focus of concerted public health control efforts based on screening and treatment [1, 2]. A systematic survey of chlamydia control activities conducted recently in 28 European Economic Area (EEA) countries [3] found wide variation in the organisation of chlamydia control: 60% (17/28) of countries reported no organised activity, while 39% (11/28) of countries had a strategy or plan for sexually transmitted infections (STI) control in place (in 6 of those 11 chlamydia control was specifically mentioned in the strategy or plan). In 2012, clinical guidelines for chlamydia case management were available in 79% of these countries (22/28); including guidelines specifically for general practice or primary care physicians (GP) in 46% of the countries (13/28) [3]. Opportunistic testing of asymptomatic individuals was recommended in guidelines of 39% of countries (11/28) but the UK is the only country with an organised chlamydia screening programme after the Netherlands stopped its pilot programme in 2012 [3].

Effective diagnosis and treatment of people with symptoms suggestive of chlamydia, followed by tracing and treatment of their recent sexual partner (s) is good clinical practice at the individual level [4]. However as the majority of infected people are asymptomatic and partner notification is often incomplete infected individuals can continue to transmit chlamydia to re-infect an existing partner, or to infect a new sexual partner. Therefore, if used alone symptomatic case management is unlikely to have an impact on chlamydia prevalence or long-term complications at the population level. Approaches for chlamydia control at the public health level include primary prevention activities, increased access to testing and treatment for people with or at risk of infection, and screening either opportunistically or as part of an organized population screening program. The effectiveness and the best approach to screening (where and whom to screen, how often, and what level of screening uptake is required to reduce prevalence in practice) is not yet clear.

A diversity of services might be required to provide comprehensive access to patients with, or at risk of, infection. General practitioners are potentially significant providers for chlamydia testing and screening [5–7]. Especially acknowledging the recent technological developments (e.g. nucleic acid amplification test (NAAT) based testing of non-invasive genital specimens) and wider access to adolescent/young adult population groups than for many other medical specialists [8–10].

The objectives of this study were to describe and compare chlamydia testing provision in four selected European countries with well-developed primary health care systems and high (reported) chlamydia rates; we aimed to compare contrasting countries where chlamydia testing is provided by GPs (England, Sweden) with countries where primary care chlamydia testing is absent or very limited (France, Estonia).

### Organisation of primary care/general medicine in participating countries

#### England

Health care in the United Kingdom (UK) has been centrally funded through the National Health Service (NHS). The NHS provides both primary and specialist health care which is largely free at the point of delivery. Recent reforms include a wide range of national quality improvement initiatives and a pay for performance scheme that accounts for around 25% of family practitioners’ income. General practitioners are responsible for registered populations of patients. The practices staffing varies in size from single general practices (GPs) to large practices with 20 or more staff (typically working in groups of 4–6 self-employed physicians). They hire nurses and a range of other ancillary staff, and act as gatekeepers to most specialist care [11]. Patients may also directly access specialist genito-urinary medicine, out of hours primary care and emergency services which are usually based in the hospital setting. Practice list sizes therefore vary between ~500 to 20,000 or more.

The core general practitioner (GP) contract is managed by NHS England, and additional public health services including sexual health are commissioned by local authorities. Some local authorities offer additional payments for an enhanced level of service provision. Patients can choose to register with any practice that covers the area they are resident in and everyone can access NHS GP services without charge. Certain services which are not considered necessary or cost effective such as travel vaccinations, cosmetic and minor surgery are not provided on the NHS.

#### Estonia

The Health Services Organization Law of 2002 established the regulatory framework for primary care and family medicine whereby primary care is organized as the first level of contact with the health system and is provided by independent general practitioners (in Estonia: family physicians) –– who are contracted by the Estonian Health Insurance Fund and paid according to the number of patients registered on the list (according to a weighted per capita payment system) and fee-for-service. Since 2006, basic allowances have been complemented by a quality bonus system, which aims to foster disease prevention and management of selected chronic conditions. The regulations specify that a practice list size should be around 1600 ± 400 persons. Patients are free to choose the general practitioner with which they register, and can change their GP when they wish. All GPs are required to work with at least one practice nurse (in order to receive payment from the Estonian Health Insurance Fund). The income from the Estonian Health Insurance Fund is used by the family physician to meet the practice running costs, purchase of equipment, cost of essential diagnostics, and for remuneration of employed staff (such as nurses or administrative staff). In Estonia, GPs work as partial gatekeepers: referral from a GP is needed for almost all specialist care, except for (dermato)venerology, psychiatry, ophthalmology, gynaecology and traumatology in the case of acute trauma.

#### France

There is no general law defining primary care in France. Most health care providers in primary care working in private practice are regulated by a contract between the GP and the national health insurance.

Most primary care providers work on a fee for service basis. In order to be optimaly refunded, each citizen has to register with a doctor designated as their attending physician”, the patient has to attend this doctor in order to be reimbursed in case of referral to some other specialists (but not for consultation with gynaecologists, paediatricians, ophthalmologists or psychiatrists). Most people are registered with a doctor (84%), and nearly all of these are with a GP (98%). There is no capitation fee related to the contract, and no commitment of any kind either for the doctor, or the patient, and no duration for the contract (patients can change “médecin traitant” at any time). GP practices are mainly single-handed. The number of group practices is increasing but very few of them include other healthcare providers (nurses, specialists, psychotherapists) [12].

#### Sweden

Primary care, delivered by public (owned by the county councils) and private (mostly owned by companies or cooperation) primary care units in Sweden, involves services that do not require advanced medical equipment and is responsible for guiding the patient to the right level within the health system. Team-based primary care facilities with four to six GPs, and other staff categories (district nurses, nurses and often physiotherapists, occupational therapists, psychologists, and social welfare counsellors), is the most common form of primary care practice in Sweden. Ideally, each GP will have 1,500 patients registered, in fact most have 2,000 or more registered patients in care.

Payment to primary care providers is generally based on capitation for registered patients, weighted with their estimated ‘illness burden’, fee-for-service and performance-based payments [13]. Primary care in Sweden for most surgeries falls within the budget of the County Council. Either the GPs and staff are employed by the County Council or they are private with a contract with the County Council.

## Methods

The methods for collection and analysis of the data was agreed between the research team members and are presented below. The design was a cross-sectional review to collect national level data from participating countries.

### Research team

The participating researchers from 4 countries brought to the team different perspectives and expertise. The study team consisted of a university researcher in public health/dermatovenerologist (STI physician) (AU; Estonia), a general practitioner (RK; Estonia); a County Medical Officer (HF; Sweden) and a Registered Nurse for Communicable Diseases (JH; Sweden); two public health physicians (BD and PD; France), and a consultant microbiologist (CMcN; England) and a primary care researcher (EJR; England).

For data generation a structured questionnaire was developed and secondary data sources searched. A structured questionnaire developed by the research team allowed a systematic approach to analysing the chlamydia care (including testing in general practice) and the gathering of relevant data.

The rationale for this analysis and methodology were evaluated by 8 study team members and discussed during research meetings throughout the analysis development process.

### Questionnaire development

A structured questionnaire was developed by the study team and discussed at a face to face meeting in English (Additional file 1). The questionnaire asked about: the occurrence of chlamydia cases reported in participating countries; national data on the existence and content of guidelines on the management of chlamydia; details of programmes for surveillance and how data was collected; chlamydia care provision across the health service; and primary health care provision. In addition, specifically for general practice settings, the following information was obtained: volume of practice participation in chlamydia screening; how GP staff usually managed chlamydia and laboratory diagnosis, payment of GP staff for chlamydia and STI care.

The research team representatives in each country were asked to fill in the questionnaire and to ask other local experts (general practitioners, commissioners of sexual health services, public health practitioners, medical education specialists, health administrators) for help if they were uncertain of the answers themselves. Representatives were also asked to send background information, such as strategy documents and guidelines. Following the face to face meeting the questionnaire was finalised and emailed in February 2013 to the study researchers in each country. A member of the research team (AU) monitored answers, responded to questions and sent reminders. A bias in the analysis was mitigated in each country by another health professional with a national interest in sexual health (for example in England the National Chlamydia Screening Programme) or GP care provisjon (for example from Department of Family Medicine of the University of Tartu) checking the responses, and by detailed review of the data at a face to face meeting and several teleconferences and by email by the research team.

Country representatives reviewed the preliminary analysis; the data used in this report include all responses, amendments and clarifications received by September 2013.

Background data from secondary sources were collated, and included a review of published literature relating to chlamydia management in the selected countries. This analysis was supplemented by documentary analysis of published reports, key legal instruments and policy documents (European CDC reports on STI surveillance and chlamydia control [14, 15]; country specific surveillance and administrative data as necessary). To assess the publication productivity (number of publications) of countries on chlamydia research and specifically on chlamydia research in primary care for the period of 1991 to 2013 a systematic literature search was conducted in PubMed using the following search terms: all - (“chlamydia”[MeSH Terms] OR “chlamydia”[All Fields]) AND (“1991”[CRDAT]: “3000”[CRDAT]) AND “humans” [MeSH Terms] AND [country]; GP setting - (“chlamydia” [MeSH Terms] OR “chlamydia”[All Fields]) AND (“1991”[CRDAT]: “3000”[CRDAT]) AND “humans”[MeSH Terms] AND “general practice”[MeSH Terms] OR (“general”[All Fields] AND “practice”[All Fields]) OR “general practice”[All Fields] AND [country].

The study team conducted systematic debriefings and cross-comparisons of the data compiled and its interpretation. Discrepancies between the researchers in interpretation were followed by in-depth discussion about the observations that led to those conclusions and additional data collection or consultations until the discrepancy was resolved.

The analysis presented here is based on the use of data or records that contain only non-identifiable data about people, e.g. publicly accessible records, archives or publications. The ethics committee of the University of Tartu has waived the requirement for ethics approval as this does not constitute research on human subjects. The study procedures were in accordance with local data protection regulations in all participating countries.

## Results

### Occurrence of chlamydia in study countries

Data on the incidence (number of reported cases in the population) and prevalence of genital chlamydia infection based on population-based studies is presented in Table 1. Sweden has the highest rate of chlamydia infections per 100,000 reported by surveillance. Availability of surveillance data from France is limited. According to the rate of new cases among 18–24 year olds in France (240-360/100,000), the estimated rate for all ages in France is likely to be similar to that reported from Estonia, rather than that in the England or Sweden. Chlamydia prevalences (derived from population based studies) range from 3.2% (France) to 6.2% (England) for women and 2.5% (France) to 5.3% (England) for men [16–19]. In the population-based studies, the gender differences of chlamydia prevalence in these countries were insignificant.

**Table 1:** **Occurrence of genital chlamydial infection and research on chlamydia in study countries**

	England	Estonia	France	Sweden
Number of reported cases, all age groups, 2010	215 501 (206 912 in 2012)	1 686 (1 596 in 2012)	Data not available	36 800 (37 700 n 2012)
Rate per 100 000 population, all age groups, in 2010 (and 2012)	348/100 000 (1979/100 000 in 2012*)	126/100 00 (119/100 000 in 2012)	360 for women 240 for men (aged 18–24, in 2006)**	391/100 000 (395/100 000 in 2012)
Trend (chlamydia rate per 100 000 in 2006) [14]	Increasing (190/100 000)	Decreasing (188/100 000)	NA	Increasing (360/100 000)
Chlamydia trachomatis prevalence (population-based studies)	6.2% in women and 5.3% in men [17] 4.5% in women and 4.4% in men [18]	6.0% in women and 4.0% in men [19]	3.2% in women and 2.5% in men [16]	No data
Research on chlamydia: number of publications referenced in PubMed, 1991-current (all publications on chlamydia/publications on chlamydia where GP settings are specified or mentioned (% of all))	1092/ 85 (7.8%)	16/ 1 (6.3%)	343/ 4 (1.2%)	460/5 (1.1%)

*Data for 2012: England - Chlamydia Testing Activity Dataset 2012 (http://www.chlamydiascreening.nhs.uk/ps/data.asp); Estonia – Health board (http://www.terviseamet.ee/nakkushaigused/nakkushaigustesse-haigestumine.html); Sweden - Swedish Institute for Infectious Control.

**Extrapolated from the Goulet V et al. 2011 [32].

Only a small fraction of the research on chlamydia has focused on care issues in general practice settings (<8% of the total research production on chlamydia in England; ~1% in France and Sweden).

### Chlamydia care and management in participating countries

There are written guidelines for health professionals (not necessarily primary care) covering diagnosis and case management of chlamydia in three of the four countries (England [7, 20], Sweden [21], Estonia [22]. In France, there are recommendations published by the national health authority for chlamydia laboratory diagnostics and screening [23, 24]. In these guidance documents chlamydia screening (i.e. testing of asymptomatic individuals) is recommended in all four countries for selected target groups: youth (England, France), pregnant women (Sweden, Estonia) and those exhibiting high risk sexual behaviour (England, Sweden, France). Retesting after positive chlamydia test (chlamydia treatment) is recommended at 3 months for all positive tests to check for repeat infection (in England), or for test of cure (in Sweden, Estonia). Partner notification and testing is also advocated in the chlamydia care guidelines for all health care providers (Estonia, France), or in guidelines for sexually transmitted infections/genitourinary medicine (STI/GUM) clinics (England). Sweden has specific partner notification guidelines [25]. Laboratory-based (England) and provider-based (Estonia, Sweden) infectious diseases surveillance systems for chlamydia are in place in three countries (England, Sweden, Estonia). A national screening program is in operation in England (Table 2) and has been pilot tested in France as part of a recent campaign by the Health Education Institute (the results from the campaign have not yet been made public).

**Table 2:** **Chlamydia management and control activities in study countries**

	England	Estonia	France	Sweden
Written guidelines or recommendations about chlamydia diagnosis and case management	UK National Guideline for the Management of Genital Tract Infection with *Chlamydia trachomatis* (*2006*)	Estonian Union against Sexually Transmitted Infections (EUSTI), 2011	2010: Health authority recommendations on laboratory diagnostic procedure only	The Board of County Medical Officers 2010
	British Association for Sexual Health and HIV (BASHH), 2006			
	National Chlamydia Screening Programme Core Guidance 6th Edition 2012			
Guidelines recommend testing for asymptomatic people	Yes	Yes	Yes	Yes
			Since 2003: recommendations for screening at- risk women aged 15–25 [32]	
Repeat test recommended for patients with a positive chlamydia test	Repeat test of positives 3 months after diagnosis	Repeat test of positives 3 months after diagnosis	No	Test for cure
Additional testing for other STIs or HIV recommended for those with positive chlamydia test	Yes	Yes	Yes	Yes
	Including HIV	Including HIV		
Partner notification recommended	Yes (patient and provider referral)	Yes (patient referral)	Yes (patient referral)	Yes (patient and provider referral)
Written guidelines or recommendations about chlamydia diagnosis and case management specifically for GPs	Yes, contained within National Chlamydia Screening Programme Core Guidance 6th Edition 2012 and BASHH 2006	No	No	Yes, Recommendations from the Board of County Medical officers, 2010
	Royal College of General Practitioners 2013 Sexually Transmitted Infections in primary care guidelines			
National chlamydia screening program	Yes	No	No	No
National surveillance data for chlamydia routinely reported	Yes	Yes	No (Not routinely, but intermittently)	Yes
Chlamydia cases reported by	Laboratories	Settings in which they are diagnosed (= physicians)	Surveillance network of volunteer (not compulsory, not reimbursed) laboratories that report detection rates of chlamydia (ReNaChla)	Settings in which they are diagnosed (= physicians)
Settings providing chlamydia testing (GUM = genitourinary medicine clinic; STI = sexually transmitted infections clinics; GYN = gynecology clinics)	GUM, sexual and reproductive health services, GPs, pharmacies, termination of pregnancy providers, internet based services, other (including targeted youth services)	Youth Health Centres; GYN; STI; internet based self-sampling;	GP; STI, GYN; Family planning clinic; Internal medicine specialist	GP; STI, GYN; ER; internet based self-sampling; Family planning clinic; Youth Health Centres
Main site for chlamydia testing	Men/women - GUM, primary care settings including GP	Women - GYN;	Youth - free and	Women: Family planning
		Men – STI clinic	anonymous family	or GP Men: STI clinic or GP
		Youth – Youth health centres	planning clinics	Youth - Youth surgeries
Breakdown of chlamydia cases by setting for diagnosis or treatment (top 3)	STI/GUM 29%	STI 20%	Presumed to originate	STI 25%
	GP 18%	GP 6%	mainly from Youth Health	GP 10%
	Family planning 15%	Youth Health Centres 30%	Centres and STI, Family	Youth Health Centres 40%
	Other 33%	GYN 30%	planning clinics	

Delivery of chlamydia care is spread between several health care settings: STI/GUM clinics (England; main site for men in Sweden, and in Estonia), gynaecology services (main site for women in Estonia), general practitioners (Sweden, England) and youth/family planning clinics (France, youth in Estonia, Sweden) (Table 2). Internet-based self-sampling is also available in England (a free service, FreeTest Me, is available), Sweden (a free service in the majority of counties), and Estonia (not free of charge).

### Primary care provision and chlamydia care in participating countries

There are some clear similarities in the structure of GP care provision in England, Estonia and Sweden. Small group (2–3 GP) practices are the typical setting for primary care provision, although in England, a sizeable proportion of practices are larger (38% - medium 5–9 GPs). In contrast, the typical GP service in France would be a single-handed practice. In England, Sweden and Estonia, nurses work in GP practices, but not in France (Table 3). Over two thirds of the youth at target age (16-24 years old) visit their general practitioner at least once a year [26–28].

**Table 3:** **Primary health care provision indicators in study countries**

	England	Estonia	France	Sweden
Number of general practitioners premises/surgeries	8230 in England	802	59 838 GPs registered in private practices	5000
	70 per 100 000	62 per 100 000	92 per 100 000	53 per 100 000
Main type of GP services	Small group practices: typically 2–3 GPs and nurses (45%); larger ones 5–9 GPs and 3–4 nurses (38%)	Single handed practices (70%); small group practices (20%); 1–2 nurses	Small group practices (54%), others single handed.	Medium size practices: 4–5 GPs, 10 nurses, 2 lab technicians, one social worker
			No nurses	
Is training for sexually transmitted disease care (during residency) mandatory for GPs?	Not mandatory, but GPs studying it must meet key competencies	Yes (but extremely limited)	No	No, but often done
Proportion of people aged 16–24 visiting GP per year	75% female, 63% male% [26]	71% [27]	75% [28]	60%*

*Local County Statistics (in Swedish).

The official policy of GP training in STI care (including chlamydia) varies in the 4 countries – from no training during GP residency (France), training some GPs but not all (England), to some training offered to all GPs (Sweden, Estonia), whether formally required (Estonia) or not (Sweden) (Table 3).

Provision of chlamydia care (testing, treatment, counselling) in GP services is regularly done in England and Sweden however in Estonia and France only a small proportion of GP practices provide chlamydia care (Table 4). For men self-taken specimens (urine) and for women physician-collected (cervical) swabs are the sampling methods of choice (with an exception of Sweden where for women self-collected vaginal swabs are used, and England where women are either asked to use a self-collected vaginal swab or urine test). For genital chlamydia infection testing, urine is generally collected from men for both symptomatic disease and screening. For women, physician-collected cervical swabs are advised for symptomatic cases and self-collected vaginal swabs for screening purposes (Table 4).

**Table 4:** **Chlamydia care in GP practices in study countries**

	England	Estonia	France	Sweden
GPs regularly provide STI care including chlamydia	Yes	No	Yes, usually only for symptomatic cases	Yes
% of GP services performing any testing for genital CT infection	88%	< 5%	< 10%	>80%
Do GP services provide screening (i.e. testing asymptomatic cases for chlamydia)?	Yes	No	No	Yes
Do GP services test symptomatic cases for chlamydia?	Yes	No	No	Yes (on the request of the patient)
Most commonly used method for chlamydia testing in GP settings	NAAT*	NAAT	NAAT	NAAT
Specimens usually used by GPs for diagnosis of symptomatic cases	Men urine; Women – physician collected cervical swab	Men – urine or physician collected urethral swab; Women – physician collected cervical swab	Men urine; Women – physician collected cervical swab	Men urine; Women – cervical swab or (self-collected) vaginal swabs
Specimens usually used by GPs for screening tests	Men urine; Women – (self-collected) vaginal swabs or urine	Men – urine or physician collected urethral swab; women – physician collected swab (endocervical) or urine	Men - urine samples: women - physician collected swab (endocervical)	Men urine; Women – (self- collected) vaginal swabs

*Nucleic acid amplification tes.

### Payment for chlamydia care in GP settings

Payment of health care services for the diagnosis and treatment of individuals with chlamydia for GPs varies in the study countries (Table 5). National insurance (Estonia) or a National health managing system (England, Sweden) reimburses the costs to GPs. In these countries, GPs are expected to provide most health care (including chlamydia care) within a particular budget per patient. Importantly there are local drivers that potentially modify chlamydia testing in general practices. In England, a diagnosis rate for chlamydia is included in the Public Health Outcomes Framework [29], used by sexual health commissioners to plan work aimed at achieving recommended testing rates (2,300 per 100,000 population) [30]. In response to that, some Local Commissioners in England choose to incentivise GPs to screen more patients by paying them to reach diagnostic targets, and/or encourage GPs to screen by stressing the importance of screening as a public health measure.

**Table 5:** **Payment for chlamydia and STI care in GP settings**

	England	Estonia	France	Sweden
Are any of the costs of [health care services for the diagnosis and treatment of individuals with chlamydia] covered or reimbursed to the GP service by the national health insurance system or its equivalent?	Yes	Yes	Yes, indirectly	Yes
	National Health Service	Estonian health insurance fund	Patients pay for directly to GPs for chlamydia care, and are afterwards (partially) reimbursed by the National health insurance	County Council
Are the costs of […] covered or reimbursed to patients?	Yes	Yes, partially (drugs up to 50%)	Yes, partially (all services up to 65%)	Yes

In Estonia, health care service costs are covered by National health insurance. The limited budget leads to prioritisation of resources to other areas as STI care is generally a low priority for GPs.

In Sweden, chlamydia care at GP level is provided within the capitation for registered patients, i.e. covered by the County Council. Further, some counties provide chlamydia diagnostic tests free of charge to the GP (e.g. Örebro County but not Uppsala County). The patients can go in every county to any GP for testing and treatment without incurring any cost.

In France patients pay GPs and laboratories directly for services rendered and afterwards are partially reimbursed by the National health insurance system. However, only 65% of the cost for opportunistic testing is reimbursed to patients belonging to risk groups only (although these risk groups are not specified). Testing is free for patents in STI, family planning and antenatal clinics.

In Estonia, 50% of the STI treatment medication costs are reimbursed to patients, and in England, patients do not have to pay for the medication if they are treated by a Genitourinary Medicine clinic, they are in full time education and under 18 years of age, in receipt of certain benefits, or are prescribed treatment under a Patient Group Direction.

## Discussion

Our aim was to compare chlamydia care in countries where testing is provided by most GP practices (England, Sweden) with that in countries where GP testing is not provided or is provided only to a very limited extent (France, Estonia) to delineate factors that may be related to the future scope and volume of chlamydia care provided by GPs.

We found little significant difference in the burden of the disease (genital chlamydia infection) or the organization of GP care provision. In all four countries, a substantial proportion (~70%) of population at most risk of chlamydia (young people aged 16–25 years) annually visit GPs. Importantly, in all the countries examined, testing for chlamydia is available in the public sector, and using NAAT methodology (NAAT is convenient, logistically straightforward, facilitates sampling, and highly specific/sensitive). An important similarity was that chlamydia care costs are reimbursed to the relevant parties (GPs in England, Sweden and Estonia; and patients in France) by the National health insurance system or its equivalent in participating countries (although not necessarily all the costs).

The countries where GPs provide chlamydia care regularly (England, Sweden) have written chlamydia management guidelines developed specifically for GPs, and STI management was more firmly established in the GP training curriculum either formally (England) or informally (Sweden). In comparison, in France, training for sexually transmitted disease care during GP training was not mandatory and in Estonia training was extremely limited (although formally in place).

A prerequisite for effective chlamydia control is the existence of a comprehensive and effective control programme, with a broad consensus on a strategy for chlamydia control, taking into account specific national opportunities and limitations, and addressing available and future services and resources. National strategies or plans on STI control that specifically mention chlamydia are in force in England and Sweden. England is the only country in the European Union implementing a national chlamydia screening program that offers opportunistic screening for chlamydia to women and men under 25 years of age attending clinical and non-clinical screening venues using non-invasive urine or vulvo-vaginal swab samples tested via NAAT [31]. In addition and in relation to that, England has specific chlamydia diagnostic targets for local authorities to reach and therefore this incentivises them to take forward chlamydia Screening Programmes with GPs.

The historical provision of STI care influences current GP staff attitudes and confidence to provide chlamydia testing in the primary care setting. In all countries the majority of STI diagnostic tests have been undertaken in the specialised genito-urinary medicine or youth/family planning of gynaecology settings. And indeed this continues to be the major provider of chlamydia testing, but we contend that the GP practice could play a much greater role. Chlamydia screening as a national program has only been undertaken in England since 2003 and is not yet embedded as routine [32]. In Estonia and France it may be difficult to change long-term traditions, especially when the special training is limited. An intervention study conducted in the South West of England was successful in increasing chlamydia testing rates in general practice through providing training, resources and support to GP staff [33]. The results of this study were intended to inform the adaptation of that intervention to implement a chlamydia testing training package for with general practice staff in England, Estonia, France and Sweden to ensure cultural and logistical issues were addressed in its development.

A recent review on potential advantages of chlamydia screening concluded that young people are generally positive about chlamydia screening, and general practice could be one of their preferred venues [34].

Several studies assessing care provision in GP surgeries have documented substantial barriers for the delivery of preventive services – such as lack of time during the visit, inadequate insurance reimbursement, belief that patient will not wish to discuss or comply with recommendations, and lack of physician expertise in counselling techniques [35]. Consistent with the finding that time is a salient barrier, Zyzanski and colleagues have shown that high-volume physicians perform fewer preventive services [36]. A study from the USA, has shown that to fully satisfy the US Preventive Services Task Force recommendations, 1,773 hours of a physician’s annual time, or 7.4 hours per working day, is needed for the provision of preventive services [35]. This confirms that time may well be a major barrier to public health initiatives in primary care and emphasises the need for prioritising interventions to be delivered and any intervention to be quick and easy.

Our study has limitations. A significant limitation is that we did not examine individual features of primary prevention present in the study countries. Further, we only assessed a small number of countries, and we are not able to generalise our results widely. However, we believe that this analysis has provided useful insights in an area where research is limited.

## Conclusions

In countries where chlamydia testing is provided by GPs (England, Sweden) a national strategy or plan on STI control that specifically mentions chlamydia was in force, chlamydia care guidelines for GPs were in place and STI management was more firmly established in the GP residency training curriculum, either formally (England) or informally (Sweden), than in the other countries.

Future research on the effectiveness of chlamydia screening (also in the context of general practice care) and program provision should reflect national needs and the prevention of complications. Our findings indicate that to attain any increase in the provision of testing for and diagnosis of STIs in the general practice setting countries with low chlamydia and STI testing will probably need nationally agreed guidance, nationally agreed STI training within the GP residency curriculum, and much on-going education support and finances within the general practice setting. We are now developing an educational resource that can be implemented across countries with different chlamydia and STI testing provision in the GP setting (http://www.STItraining.eu).

## Electronic supplementary material

Additional file 1: **Questionnaire used for study data collection.** (XLS 57 KB)

## References

[1] European Centre for Disease Prevention and Control (2012). Annual epidemiological report Reporting on 2010 surveillance data and 2011 epidemic intelligence data.

[2] Centers for Disease Control and Prevention (2011). Sexually Transmitted Disease Surveillance 2010.

[3] European Centre for Disease Prevention (2013). (Draft) technical report: Chlamydia Control in Europe Survey of Member States.

[4] European Centre for Disease Prevention and Control (2009). GUIDANCE Chlamydia control in Europe.

[5] Shaw K, Coleman D, O’Sullivan M, Stephens N (2011). Public health policies and management strategies for genital *Chlamydia trachomatis* infection. Risk Manage Healthc Policy.

[6] Kalwij S, French S, Mugezi R, Baraitser P (2012). Using educational outreach and a financial incentive to increase general practices’ contribution to chlamydia screening in South-East London 2003–2011. BMC Public Health.

[7] *National Chlamydia Screening Programme Standards*. 6th edition. 2012. available at http://www.chlamydiascreening.nhs.uk/ps/resources/core-requirements/NCSP%20Standards%206th%20Edition_October%202012.pdf

[8] Guy RJ, Ali H, Liu B, Poznanski S, Ward J, Donovan B, Kaldor J, Hocking J (2011). Efficacy of interventions to increase the uptake of chlamydia screening in primary care: a systematic review. BMC Infect Dis.

[9] Ginige S, Fairley CK, Hocking JS, Bowden FJ, Chen MY (2007). Interventions for increasing chlamydia screening in primary care: a review. BMC Public Health.

[10] McNulty CA, Thomas M, Bowen J, Buckley C, Charlett A, Gelb D, Foy C, Sloss J, Smellie S (2008). Interactive workshops increase chlamydia testing in primary care–a controlled study. Fam Pract.

[11] Roland M, Guthrie B, Thome DC (2012). Primary Medical Care in the United Kingdom. J Am Board Fam Med.

[12] Samuelson M, Bourgueil Y (2008). Country Case Study: Primary care in France. Presentation at the Conference Improving primary care in Europe and the US.

[13] Glenngård AH, Hjalte F, Svensson M, Anell A, Bankauskaite V (2005). Health systems in transition.

[14] European Centre for Disease Prevention and Control (2013). Annual Epidemiological Report 2012. Reporting on 2010 surveillance data and 2011 epidemic intelligence data.

[15] European Centre for Disease Prevention (2008). Technical report re view of chlamydia control activities in EU countries.

[16] Goulet V, de Barbeyrac B, Raherison S, Prudhomme M, Semaille C, Warszawski J (2010). CSF group: Prevalence of Chlamydia trachomatis: results from the first national population-based survey in France. Sex Transm Infect.

[17] Low N, McCarthy A, Macleod J, Salisbury C, Campbell R, Roberts TE, Horner P, Skidmore S, Sterne JA, Sanford E, Ibrahim F, Holloway A, Patel R, Barton PM, Robinson SM, Mills N, Graham A, Herring A, Caul EO, Davey Smith G, Hobbs FD, Ross JD, Egger M, Chlamydia Screening Studies Project Group (2007). Epidemiological, social, diagnostic and economic evaluation of population screening for genital chlamydial infection. Health Technol Assess.

[18] Bracebridge S, Bachmann MO, Ramkhelawon K, Woolnough A (2012). Evaluation of a systematic postal screening and treatment service for genital Chlamydia trachomatis, with remote clinic access via the internet: a cross-sectional study, East of England. Sex Transm Infect.

[19] Uusküla A, Kals M, Denks K, Nurm U, Kasesalu L, Dehovitz J, McNutt LA (2008). The prevalence of chlamydial infection in Estonia: a population-based survey. Int J STD AIDS.

[20] British Association Sexual Health and HIV 2006 (2006). UK National Guideline for the Management of Genital Tract Infection with Chlamydia trachomatis.

[21] *Sweden STI management guidelines*. available at http://www.slf.se/Foreningarnasstartsidor/Intresseforening/Smittskyddslakarforeningen/Smittskyddsblad-/Klamydialakarinformation-2010-02-15/, in Swedish

[22] Estonian union for sexually transmitted infections (2011). STI management guidelines 2011.

[23] *Haute autorite de sante 2010 Diagnostic biologique de l’infection à Chlamydia trachomatis*. 2012. http://www.has-sante.fr/portail/upload/docs/application/pdf/2012-02/synthese_chlamydia_trachomatis.pdf

[24] Haute autorite de sante 2003 (2003). Evaluation du depistage des infections uro-genitales basses à chlamydia trachomatis en France.

[25] Smittspårning vid sexuellt överförbara sjukdomar (2007). Recommendations on partner notification.

[26] Salisbury C, Macleod J, Egger M, McCarthy A, Patel R, Holloway A, Ibrahim F, Sterne JA, Horner P, Low N (2006). Opportunistic and systematic screening for chlamydia: a study of consultations by young adults in general practice. Br J Gen Pract.

[27] Tekkel M, Veideman T (2012). Health Behavior among Estonian Adult Population, 2012. Natl Inst Health Dev.

[28] Beck F, Richard JB (2013). Les Comportements de santé des jeunes. Analyses du Baromètre santé 2010. Saint-Denis: Inpes, coll. Baromètres santé.

[29] PHPSU/HIAT/12341 (2013). Public Health Outcomes Framework.

[30] *Public Health Outcomes Framework 2013 to 2016 and technical updates*. England: Department of Health; available at https://www.gov.uk/government/publications/healthy-lives-healthy-people-improving-outcomes-and-supporting-transparency

[31] *National Chlamydia Screening Programme*. http://www.chlamydiascreening.nhs.uk/

[32] Dehne KL, Riedner G, Neckermann C, Mykyev O, Ndowa FJ, Laukamm-Josten U (2002). A survey of STI policies and programmes in Europe: preliminary results. Sex Transm Infect.

[33] McNulty CA, Hogan AH, Ricketts EJ, Wallace L, Oliver I, Campbell R, Kalwij S, O’Connell E, Charlett A (2013). Increasing chlamydia screening tests in general practice: a modified Zelen prospective Cluster Randomised Controlled Trial evaluating a complex intervention based on the Theory of Planned Behaviour. Sex Transm Infect.

[34] Perkins E, Carlisle C, Jackson N (2003). Opportunistic screening for Chlamydia in general practice: the experience of health professionals. Health Soc Care Community.

[35] Yarnall KS, Pollak KI, Østbye T, Krause KM, Michener JL (2003). Primary care: is there enough time for prevention?. Am J Public Health.

[36] Zyzanski SJ, Stange KC, Langa D, Flocke SA (1998). Trade-offs in high-volume primary care practice. J Fam Pract.

[CR37] The pre-publication history for this paper can be accessed here:http://www.biomedcentral.com/1471-2458/14/1147/prepub

